# Computational characterization and identification of human polycystic ovary syndrome genes

**DOI:** 10.1038/s41598-018-31110-4

**Published:** 2018-08-28

**Authors:** Xing-Zhong Zhang, Yan-Li Pang, Xian Wang, Yan-Hui Li

**Affiliations:** 1 Department of Physiology and Pathophysiology, School of Basic Medical Sciences, Peking University, Beijing, China; 2 Institute of Cardiovascular Sciences, Peking University, Beijing, China; 3 Department of Obstetrics and Gynecology, Center for Reproductive Medicine, Peking University Third Hospital, Beijing, China

## Abstract

Human polycystic ovary syndrome (PCOS) is a highly heritable disease regulated by genetic and environmental factors. Identifying PCOS genes is time consuming and costly in wet-lab. Developing an algorithm to predict PCOS candidates will be helpful. In this study, for the first time, we systematically analyzed properties of human PCOS genes. Compared with genes not yet known to be involved in PCOS regulation, known PCOS genes display distinguishing characteristics: (i) they tend to be located at network center; (ii) they tend to interact with each other; (iii) they tend to enrich in certain biological processes. Based on these features, we developed a machine-learning algorithm to predict new PCOS genes. 233 PCOS candidates were predicted with a posterior probability >0.9. Evidence supporting 7 of the top 10 predictions has been found.

## Introduction

Polycystic ovary syndrome (PCOS) is a highly complex disorder that affects 6–10% of women of reproductive age^1^. It is a major cause of anovulatory infertility and increases the risk for insulin resistance, obesity, cardiovascular disease and psychosocial disorders^2,3^. Studies have shown that PCOS is regulated by the subtle interaction of genes and environmental factors^4–6^.

To identify PCOS genes, reverse genetics like microarray studies have profiled whole-genome gene expression in a number of PCOS tissues, including ovary^7,8^ and adipose^9^. Genome-wide association study (GWAS) is used to identify regions of the genome that harbor variants associated with disease risk or quantitative traits^10–12^. For computational methods, a group once reconstructed transcription factor-microRNA synergistic regulatory network, and they considered the nodes with highest degree as PCOS candidate genes^13^. Another group constructed a protein-protein interaction (PPI) subnetwork and selected the top hubs (both high degree and betweenness) as PCOS candidates^14^. However, both works lack rigorous statistics to evaluate the accuracy of the prediction. To our knowledge, no efficient algorithm has been developed to predict PCOS genes. In fact, bioinformatics algorithms have been successfully developed to infer candidate genes in other fields^15–18^, and these could be introduced to PCOS research.

In this work, we developed a method to identify distinguishing properties of PCOS genes and subsequently used them to predict new candidates. We firstly systematically compared the computational characteristics of two groups of genes: known PCOS genes versus the remaining genes in the genome (called non-PCOS genes hereafter). We examined each set of the genes in network topological features and functional annotations. Then, we singled out the features with significant difference between PCOS and non-PCOS genes by Kolmogorov–Smirnov (KS) test. We employed support vector machine (SVM) with liner function as the classifier. Finally, with a posterior probability >0.9, 233 new PCOS genes were predicted. Literature supporting 7 of the top 10 predictions has been found.

## Results

### PCOS genes tend to have higher degrees

**Figure 1:**
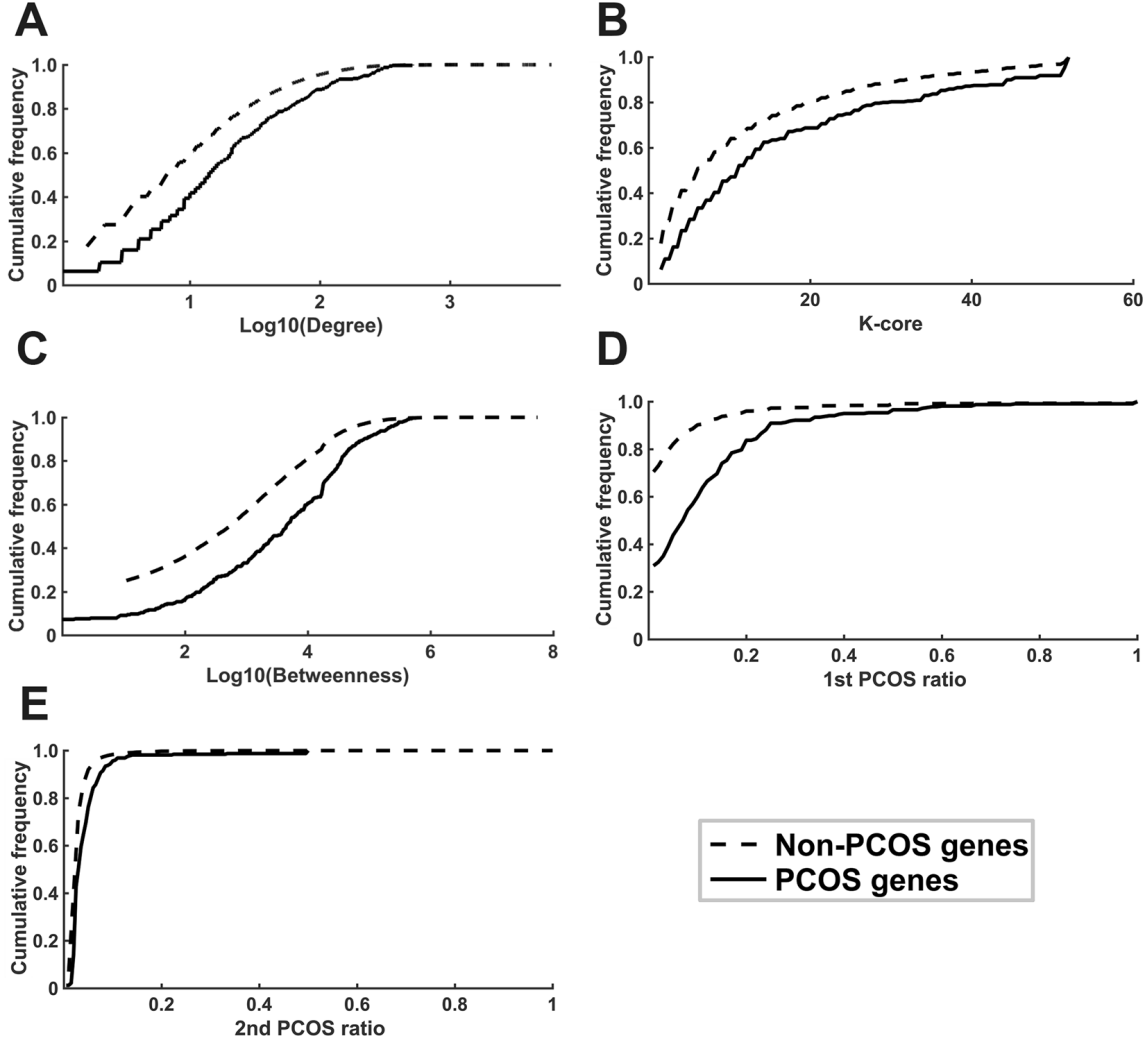
Cumulative frequency distributions of network features of PCOS genes and non-PCOS genes. The PCOS genes tend to have higher degree (**A**), *K*-core (**B**), betweenness (**C**), 1st PCOS ratio (**D**), and 2nd PCOS ratio (**E**) than that of non-PCOS genes. The cumulative frequency of different features is 100% for PCOS genes and non-PCOS genes. PCOS represents polycystic ovary syndrome.

For a protein, its degree is defined as the number of direct interaction genes. According to network theory, a protein with more direct interaction neighbors (higher degree) might be more important to the network^19^. Based on PPI network downloaded from OPHID^20^, we counted the number of direct interaction neighbors for each gene, and found that PCOS genes tend to have higher degrees than non-PCOS genes. The average degrees for PCOS genes and non-PCOS genes are 41.81 and 22.48, respectively (see Table 1). The cumulative frequency distribution curves of degrees for PCOS genes shift to the right compared with that of non-PCOS genes (Fig. 1A**)**. There is significant difference between them, with *P* = 4.2E-13 by KS test.

**Table 1:** Network Characteristics of PCOS Genes.

Dataset	Class	Size	Degree	*K*-Core	Betweenness	1st PCOS Ratio	2nd PCOS Ratio
Total	PCOS	306	41.81	16.78	34463	0.11	0.04
	Non-PCOS	16676	22.48	11.66	17278	0.04	0.03
	*P* value		4.2E-13	1.7E-10	1.4E-17	3.0E-48	6.0E-20
PCOSDB	PCOS	185	50.54	18.78	40177	0.10	0.03
	Non-PCOS	16676	22.48	11.66	17278	0.02	0.02
	*P* value		1.5E-12	5.2E-10	2.0E-12	2.0E-36	1.3E-09
PCOSKB	PCOS	226	38.60	15.43	33614	0.09	0.04
	Non-PCOS	16676	22.48	11.66	17278	0.03	0.02
	*P* value		2.6E-07	9.1E-07	5.3E-12	1.5E-32	1.1E-20

“Total” indicates all the PCOS genes covered by either PCOSDB or PCOSKB. “Non-PCOS” indicates the remaining genes. The degree of a gene is defined as the number of its direct interaction genes. A *K*-core of a network can be obtained by recursively deleting genes with a degree lower than *K*, until the remaining genes in subnetwork have a degree higher than *K*. Betweenness counts the number of times that a gene is on the shortest path between two other genes. 1st PCOS ratio is defined as the ratio of the number of PCOS genes that it direct interacts to its degree. 2nd PCOS ratio is defined as the ratio of the number of PCOS genes that belong to 2nd interaction genes to its number of 2nd interaction genes. The *P* values were calculated by KS test. PCOS represents polycystic ovary syndrome.

To validate the results, we also analyzed degrees of PCOSDB genes or PCOSKB genes separately. The average degree of PCOSDB genes is 50.54 while that from PCOSKB equals to 38.60, which are all significantly higher than that of non-PCOS genes. These results were listed in Table 1.

### PCOS genes tend to enrich at global network center

Genes with high degrees might locate at globally or locally central position, while only those located globally are more likely to be evolutionarily conserved^21^. To distinguish the different locations, we calculated *K*-core for each gene. The *K*-core gradually displays the backbone of a network by iteratively deleting genes with a degree lower than *K*, remaining genes in the subnetwork with a degree higher than *K*. If a gene has a high *K*-core, then it is more likely to be located at global center. We found that PCOS genes have an average *K*-core of 16.78, whereas the average *K*-core of non-PCOS genes is only 11.66 (Table 1). KS test showed that there is significant difference, with *P* = 1.7E-10. The cumulative frequency distribution of *K*-core values for PCOS genes and non-PCOS genes are shown in Fig. 1B. And from the other two datasets, we obtained similar results, indicating that the high *K*-core of PCOS genes is data independent (Table 1).

Betweenness is another frequently used measure of network centrality, which counts the number of shortest paths between two other genes that go through a gene of interest. Therefore, a gene with a high betweenness could be considered as a bottleneck node in the network^22^. The results showed that PCOS genes had significantly higher connectivity along the shortest path between two genes than that of non-PCOS genes, with the average betweennesses are 34,463 and 17,278 (*P* = 1.4E-17 by KS test; Table 1 and Fig. 1C). And as shown in Table 1, PCOS genes have similar average betweenness from the other two datasets.

### PCOS genes tend to interact with each other

Genes function through interaction with each other in signaling pathways, therefore, we reason that direct interaction neighbors of PCOS genes might also tend to be PCOS genes. To test this, for each gene, we calculated the 1st PCOS ratio, which is defined as a ratio of the number of PCOS genes that it directly interacts to its degree. For example, IGF1 (P05019) and IGF2 (P01344) have 16 and 21 direct interaction genes, respectively, and 9 and 12 of which are PCOS genes. The 1st PCOS ratio for IGF1 and IGF2 are 0.5625 = 9/16 and 0.5714 = 12/21. The cumulative frequency distribution of 1st PCOS ratios for PCOS genes and non-PCOS genes are shown in Fig. 1D. As shown in Table 1, the PCOS genes have an average 1st PCOS ratio of 0.11, which is significant higher than 0.04 for that of non-PCOS genes (*P* = 3.0E-48; KS test).

Meanwhile, for each gene, we also calculated the 2nd PCOS ratio, which is defined as the number of PCOS genes that belong to its 2-step interaction genes divided by the number of all its 2-step interaction genes. We found that PCOS genes have an average 2nd PCOS ratio of 0.04, which is significantly higher than 0.03, the value for that of non-PCOS genes, *P* = 6.0E-20 by KS test. There results could be found in Table 1 and Fig. 1E.

### GO functional enrichment

As reported, genes associated with the same disease are often functionally related^18,23^. To examine whether PCOS genes tend to take part in some biological processes, a log-odds score was computed for each GO term to compare the frequency at which PCOS genes and non-PCOS genes were annotated to it. The distributions of log-odds scores have a significant difference between PCOS genes and non-PCOS genes (*P* = 2.1E-66; KS test), indicating that PCOS genes tend to enrich in some biological processes.

No ovulation is a major diagnostic criterion for PCOS^2^. As shown in Supplementary Table S1, “GO:0022602 ovulation cycle process” is enriched with PCOS genes. Steroid hormone plays an important role in ovarian development and ovulation process. Consistently, “GO:0042446 hormone biosynthetic process” is enriched. Meanwhile, “GO:0045940 positive regulation of steroid metabolic process” is significantly enriched with a log-odds score of 3.57, because steroid is a precursor for steroid hormone. These results indicate dysregulation of steroid hormone might be one major cause of PCOS. PCOS is a complex metabolic disease, and insulin resistance is another etiology^1^. Thus, GO terms associated with regulation of plasma glucose are enriched, such as “GO:0048009 insulin-like growth factor receptor signaling pathway” and “GO:0010828 positive regulation of glucose transport”.

### The performance of the classifier

**Figure 2:**
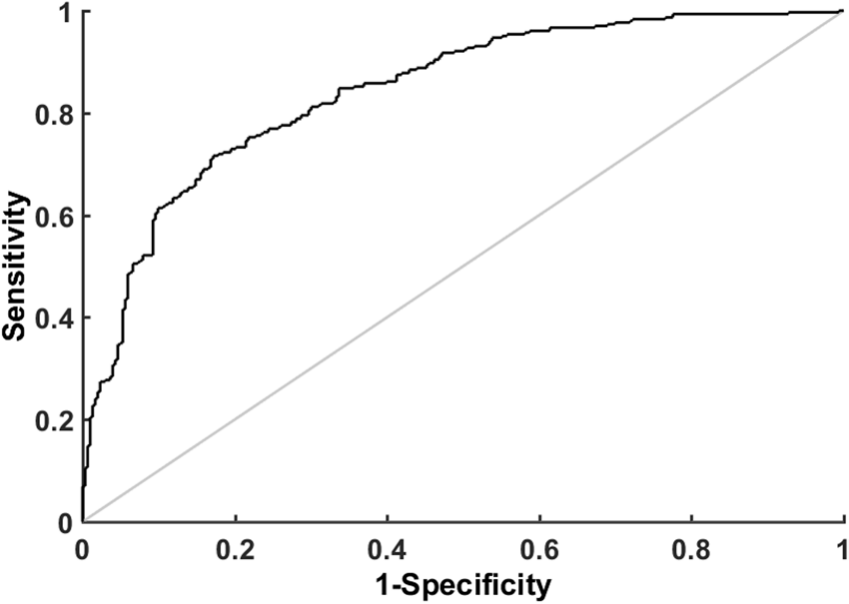
The ROC curve of SVM (liner). SVM (liner) achieved the best classification performance using network and GO functional features.

To model all above features, we tested different classifiers, *K*-nearest neighbor (KNN), decision tree and SVM with different kernel functions. As described in the Materials and methods, 306 PCOS genes downloaded from PCOSDB and PCOSKB were used as positive samples, and 306 negative genes were randomly sampled from the non-PCOS genes. Since random sampling might introduce bias, we sampled 1001 negative datasets and combined each negative dataset with the positive dataset to train the classifier. The median value of 1001 results was used to evaluate the performance of different classifiers. As shown in the Table 2, we found SVM (liner) achieved the best performance, with precision = 0.81, recall = 0.71, F1 = 0.75, and AUC = 0.80. Thus, it was chosen as the final classifier and used for real application. To show the variances introduced by sampling, the boxplots of the 1001 training results (precisions, recalls, F1s, and AUCs) with SVM (liner) are given in Supplementary Figure S1. The ROC curves of SVM (liner) could be found in Fig. 2.

**Table 2:** The Classification Performance of Different Classifiers.

Classifier	Precision	Recall	F1	AUC
KNN (*K* = 7)	0.77	0.69	0.73	0.78
Decision tree	0.76	0.74	0.75	0.79
SVM (liner)	**0**.**81**	0.71	**0**.**75**	**0**.**80**
SVM (polynomial d = 3)	0.49	0.73	0.58	0.57
SVM (RBF)	0.79	0.68	0.73	0.79

SVM (linear), SVM (polynomial d = 3) and SVM (RBF) means the kernel function of SVM is linear, polynomial, and radial basis function, respectively.

Besides cross-validation, we also tested the classifier with independent datasets. Firstly, we used the 226 PCOSKB genes as positive samples and took the 185 PCOSDB genes as test samples. We randomly selected 226 genes from non-PCOS ones as negative samples to train the classifier. After repeating 1001 times, we employed the model with median AUC value to predict the PCOSDB genes. Of the 79 PCOSDB genes (excluding the ones in the PCOSKB), 53, 32 and 15 genes were recalled with a posterior probability higher than 0.5, 0.8 and 0.9, respectively. This also showed the algorithm is helpful.

### Real application

To predict new PCOS candidate genes, we took the 306 PCOS genes from PCOSDB and PCOSKB as positive samples. And the genes in the dataset that got median AUC value of the 1001 randomly selected datasets were taken as negative samples. After training the classifier, we found that 13,681 unknown genes could be predicted by the algorithm. With a posterior probability higher than 0.9, 233 genes were predicted as PCOS genes (Supplementary Table S2). The top 25 genes are listed in Table 3.

**Table 3:** Top 25 Predicted PCOS Genes.

Symbol	Name	Posterior Probability
CTNNB1	catenin beta 1	0.99932
THBS1	thrombospondin 1	0.99864
IFNG	interferon gamma	0.99794
SMAD3	SMAD family member 3	0.99736
WNT5A	Wnt family member 5 A	0.99694
EGFR	epidermal growth factor receptor	0.9964
HIF1A	hypoxia inducible factor 1 subunit alpha	0.99623
SRC	SRC proto-oncogene, non-receptor tyrosine kinase	0.99614
ENG	endoglin	0.99536
NOG	noggin	0.99505
SIRT1	sirtuin 1	0.99498
PTEN	phosphatase and tensin homolog	0.99429
SHH	sonic hedgehog	0.9936
CAV1	caveolin 1	0.9934
SMAD4	SMAD family member 4	0.99129
GREM1	gremlin 1, DAN family BMP antagonist	0.9893
BMP10	bone morphogenetic protein 10	0.9886
GDF5	growth differentiation factor 5	0.98854
FGA	fibrinogen alpha chain	0.98846
GATA3	GATA binding protein 3	0.98752
TGFBR3	transforming growth factor beta receptor 3	0.98745
JAK2	Janus kinase 2	0.98715
LYN	LYN proto-oncogene, Src family tyrosine kinase	0.98662
NOTCH1	notch 1	0.98624
LGALS9	galectin 9	0.98621

Posterior probability is given by SVM to evaluate the reliability of the prediction. SVM represents support vector machine.

To validate our predictions, we searched literature in PubMed and found evidence for 7, 10 and 14 of the top 10, 20 and 50 genes, respectively. For example, As shown in Supplementary Table S3, CTNNB1 is predicted as PCOS gene with a posterior probability = 0.9993. A significant reduction of the expression of CTNNB1 was reported in granulosa cells from patients with PCOS compared with control group^24^. For another example, SMAD3 is predicted as PCOS gene with a posterior probability = 0.9979. Allele rs11031006-G in SMAD3 was reported to be associated with lower PCOS risk^25^.

## Discussion

In this work, we systematically investigated properties of PCOS genes and then developed an algorithm to predict new PCOS genes by integrating network characteristics and GO functional characteristics. Different from GWAS and other genetic methods, this work opens a new avenue to infer PCOS candidates.

Previously, two methods have been reported to infer PCOS genes^13,14^. One used degree as feature^13^ and the other used both degree and betweenness as features^14^. In this work, besides degree and betweenness, we considered more network topological features like *K*-core, 1st PCOS ratio and 2nd PCOS ratio. And we integrated GO functional annotations to the algorithm. More important, our method is a supervised machine-learning algorithm, with rigorous statistics to evaluate the performance. And each predicted gene has a probability to evaluate the reliability of the prediction.

According to PCOSDB and PCOSKB, both IGF1 and IGF2 are PCOS genes. And in the PPI network, we found that most of their direct interaction neighbours are also PCOS genes. In addition, gene set enrichment analysis showed that IGF receptor signalling pathway (GO:0048009) is statistically enriched by PCOS genes, in which 18 of the 36 annotated genes are PCOS genes. And 12 of the rest 18 genes were predicted as PCOS candidates by our algorithm. These results are consistent with recent researches that IGF signalling pathways might play an important role in PCOS regulation^26–28^.

It is known that PCOS is a highly heritable (70%) disease^29^. However, to date, only one gene named PCOS1 has been collected to online Mendelian inheritance in man database^30^. The PCOS genes analyzed in this work are downloaded from PCOSDB or PCOSKB. They are in fact PCOS-causing genes or PCOS-associated genes, since the causal relationships might need further confirmation by physiological studies. Here, we called them PCOS genes on one hand for short, on the other hand to highlight the importance of genetic background.

In this work, we defined 306 PCOS genes as positive samples and sampled 306 negative samples from the rest genes (13,681 = 13,987−306). We trained SVM model and evaluated classification performance with an equal number of positives and negatives, which has been widely adopted in previous studies^16,17,23^. However, as mentioned by Myers *et al*.^31^, we should carefully interpret the results based on this method, because it is achieved under the assumption that the number of positives to the number of negatives equals to 1:1 in real application domain. Notably, it would also be improper if all remaining genes were selected as negatives. Because there might be not-yet-identified PCOS genes in negative samples, which might seriously underestimate the classifier.

Notably, current PPI data is far from perfect. They usually contain a number of false positive interactions and even more false negatives. Thus, some limitations are inevitable. For example, the degree of a protein might be related to the number of researches on it. And *K*-core, 1st and 2nd PCOS ratios might be indirectly related to such research bias. We think, with the improvement of PPI data quality, these problems could be solved and our approach could be more effective.

## Materials and Methods

### Data source

The PPI data were downloaded from the Online Predicted Human Interaction Database (OPHID; http://ophid.utoronto.ca/ophidv2.204/)^20^. After deleting self-interactions and redundant interactions, the final PPI network covers a total of 16,982 proteins and 193,949 edges. Two lists of PCOS genes were downloaded from the Polycystic Ovary Syndrome Database (PCOSDB; http://www.pcosdb.net/)^32^ and the KnowledgeBase on Polycystic Ovary Syndrome (PCOSKB; http://pcoskb.bicnirrh.res.in)^33^, with 208 and 241 genes, respectively. 185 of the 208 PCOSDB genes, and 226 of the 241 PCOSKB genes were covered by the OPHID network. We combined the PCOSDB genes and the PCOSKB genes and got 306 PCOS genes in total. The functional annotations of gene products were obtained from gene ontology (GO) http://www. geneontology.org^34^. The source codes could be downloaded from Github: https://github.com/Heyuanshan/PCOS-genes-prediction.git.

### Network topological features

The network features analyzed in this work, i.e., degree, *K*-core, betweenness and PCOS ratios (1st and 2nd), are defined in Table 4. They were computed by an R package, igraph^35^.

**Table 4:** Formal Representation of Graph Measures.

Name	Function	Descriptions
Degree	${{\rm{K}}}_{{\rm{i}}}^{1}$	the number of direct interaction partners of node i
Degree-2	${{\rm{K}}}_{{\rm{i}}}^{2}$	the number of 2-step interaction partners of node i
1st PCOS ratio	${{\rm{K}}}_{{\rm{i}}}^{1,{\rm{P}}}/{{\rm{K}}}_{{\rm{i}}}^{1}$	${{\rm{K}}}_{{\rm{i}}}^{1,{\rm{P}}}$ is the number of direct interactions between node i and proteins encoded by PCOS genes
2nd PCOS ratio	${{\rm{K}}}_{{\rm{i}}}^{2,{\rm{P}}}/{{\rm{K}}}_{{\rm{i}}}^{2}$	${{\rm{K}}}_{{\rm{i}}}^{2,{\rm{P}}}$ is the number of 2-step interaction between node i and proteins encoded by PCOS genes
Betweenness	$$\sum _{\begin{array}{c}j\in V,k\in V\\ j\ne i,k\ne i\end{array}}\frac{\sigma (j,i,k)}{\sigma (j,k)}$$	$\sigma (j,I,k)$ is the total number of shortest connections between nodes j and k, where each shortest connection has to pass node i, and $\sigma (j,k)$ is the total number of shortest connections between j and k. The set V of nodes represents all proteins in the network.
*K*-core	*K*	A *K*-core of a graph can be obtained by recursively removing all nodes with a degree less than *K*, until all nodes in the remaining graph have a degree at least *K*.

Functions are the definitions of the topological features. Descriptions give explanations for symbols in the definitions.

### Log-odds score

We defined the log-odds score to describe the relative frequency with which a GO biological process was used to annotate PCOS or non-PCOS genes. The formula for calculation is as follows:

$$\mathrm{Log}\,-{\rm{odds}}-{\rm{score}}=\,\mathrm{log}(\frac{(m+a)/(n+a)}{{m}_{0}/{n}_{0}})$$

*m*_0_ is the number of PCOS genes; *n*_0_ is the total number of genes in human genome; *m* is the number of PCOS genes annotated to a GO term; and *n* is the total number of human genes annotated to the GO term. a (a = 1) is a correction factor. To avoid bias, we only used the GO terms annotated with more than 5 genes (n ≥ 5). If a gene annotated to a GO term with a high log-odds score, then the gene is more likely a PCOS gene. If a gene is annotated to several GO terms, the log-odds scores of these GO terms were summed to reflect its total associations to PCOS.

### Kolmogorov–Smirnov test

The Kolmogorov-Smirnov test is a useful nonparametric statistical method for comparing two samples through quantifying a distance between the empirical distribution functions of them. In this work, we used two sample KS test to compare the network features and functional annotations between PCOS genes and non-PCOS genes.

### Classifiers

We tested different classifiers to predict PCOS genes: *K*-nearest neighbor (KNN), decision tree and SVM with different kernel functions. KNN and decision tree were employed from MATLAB, and SVM were employed from LIBSVM3.22^36^. As shown in the Results, SVM with linear kernel achieved the best performance. The parameter *c* was optimized and set at 1. For each predicted gene, LIBSVM gives a posterior probability to evaluate its reliability^37^. If a gene gets a larger posterior probability, then it is more likely a PCOS gene.

### Positive and negative samples

The 306 PCOS genes obtained from PCOSDB and PCOSKB were used as positive samples. We randomly selected 306 genes from the rest of the human genome as the negative samples. This method has frequently been used to predict disease genes^16,17,23^. To avoid sampling bias, we sampled 1001 times of the negative datasets of 306 genes, and combined each negative dataset with the positive dataset to train the classifier.

### Classifier evaluation

As in previous works^18^, we used 5-fold cross-validation to evaluate the classifier, in which 20 percent of the whole data were left out as the test data and the remaining (80 percent) as the training data. Precision, recall, F1 score, and area under curve (AUC) were used as the measures to evaluate the classification performance. For each test dataset, we counted the numbers of true positives (TP), false negatives (FN), true negatives (TN) and false positives (FP). The formulas for calculating precision, recall, and F1 score were as following:

$$Precision=\frac{TP}{TP+FP},Recall=\frac{TP}{TP+FN},F1=\frac{2\ast Precision\ast Recall}{Precision+Recall},$$

Because we sampled 1001 negative datasets, and combined each negative dataset with the positive dataset to train the classifier, we got 1001 training results. We used the median of the 1001 values of precisions, recalls, F1s, and AUCs as the final results.

## Electronic supplementary material

Supplementary information

Dataset 1

Dataset 2

Dataset 3
