# Supplementary material for: Computational characterization and identification of human polycystic ovary syndrome genes
Source: Sci Rep. 2018 Aug 28;8:12949. doi: 10.1038/s41598-018-31110-4 (PMC6113217; doi:10.1038/s41598-018-31110-4)

**ONLINE SUPPLEMENTARY DATA**

**Computational characterization and identification of human polycystic ovary syndrome genes**

Xing-Zhong Zhang^a^ , Yan-Li Pang^c^ , Xian Wang^a,^* and Yan-Hui Li^b,^*

^a^Department of Physiology and Pathophysiology, School of Basic Medical Sciences, Peking University

^b^Peking University Health Science Center, Beijing 100191, People’s Republic of China

^c^Department of Obstetrics and Gynecology, Center for Reproductive Medicine, Peking University Third Hospital, Beijing, China

*To whom reprint request should be addressed.

Xian Wang

Department of Physiology and Pathophysiology, School of Basic Medical Sciences, Peking University

E-mail: xwang@bjmu.edu.cn

Tel: +86-10-82801443

Yan-Hui Li

Peking University Health Science Center, Beijing, China

E-mail: [liyanhui@bjmu.edu.cn](mailto:liyanhui@bjmu.edu.cn)

Tel: +86-10-82805008

**Supplementary Files**

**Supplementary Figure S1**

**Boxplots of precisions, recalls, F1s and AUCs of SVM (liner) on the 1001 training datasets**

To train classifier, positive dataset and negative dataset were required. The positive dataset were combined by PCOSDB and PCOSKB. The negative dataset were gotten by random sampling from the genome. To avoid bias that might introduce by sampling, we sampled 1001 negative datasets and combined each negative dataset with the positive dataset to train SVM (liner). Boxplot of the 1001 training results (*precision*, *recall*, *F1* and *AUC*) were shown here.

**Supplementary Table S1**

The log-odds scores of GO functions enriched by PCOS genes.

**Supplementary Table S2**

The 233 PCOS genes predicted by our algorithm.

**Supplementary Table S3**

Literature supports 14 of the top 50 predicted PCOS genes.


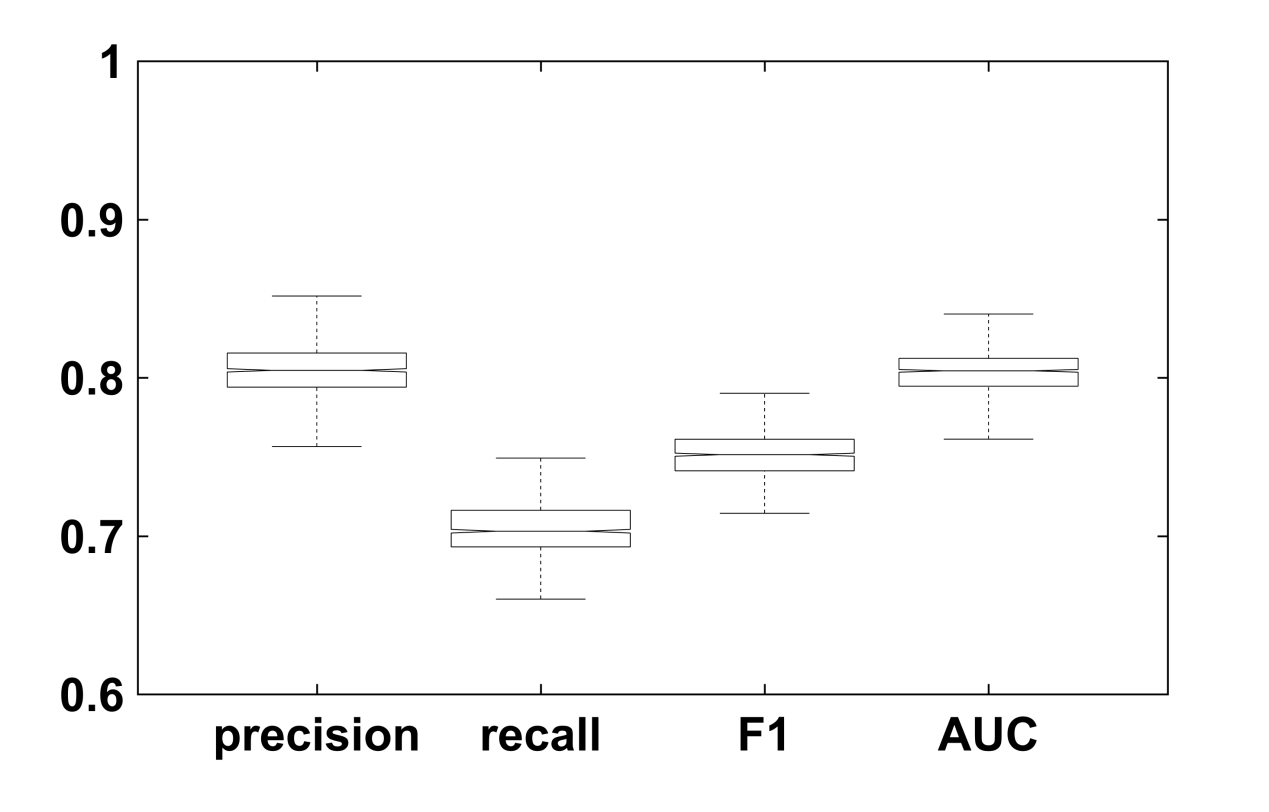

Supplement: Supplementary file 1 — Supplementary information [file 41598_2018_31110_MOESM1_ESM.docx]
